# Exploring digital health user engagement: General app usage patterns from a clinical trial with the mLab App

**DOI:** 10.1371/journal.pdig.0001452

**Published:** 2026-06-25

**Authors:** Thomas F. Scherr, Austin Hardcastle, Carson P. Moore, Dheemanth Majji, Lisa M. Kuhns, Robert Garofalo, Rebecca Schnall

**Affiliations:** 1 Department of Chemistry, College of Arts and Science, Vanderbilt University, Nashville, Tennessee, United States of America; 2 Division of Adolescent & Young Adult Medicine, Ann & Robert H. Lurie Children’s Hospital of Chicago, Chicago, Illinois, United States of America; 3 Feinberg School of Medicine, Northwestern University, Chicago, Illinois, United States of America; 4 School of Nursing, Columbia University, New York, New York, United States of America; Iran University of Medical Sciences, IRAN, ISLAMIC REPUBLIC OF

## Abstract

The number of digital health applications has rapidly grown over recent years, but many of them are limited by sustainability and scalability. Paradata, the detailed interactions of a user with a piece of software, is straightforward to collect without disrupting the user-experience, and can provide a nuanced understanding of in-app user behavior. In this work we analyze paradata from the mLab App arm of a three-arm, multi-site randomized clinical trial (NYC and Chicago), in a sub-study that evaluates longitudinal, session-level interaction logs with multiple sessions per participant, (registered with Clinicaltrials.gov as NCT03803683), a mobile health application to facilitate at-home HIV testing in at-risk populations. We investigated application-level usage statistics to identify feature usage, as well as common navigational paths within the application. Temporal patterns were observed for login events and test-taking patterns. Despite being under-collected and underreported, paradata reveal feature gaps, guide targeted revisions for subsequent research and implementation, and deepen understanding of user behavior—enabling digital-health applications with lasting impact.

## Author summary

Smartphone and web-based health apps are an increasingly common way to deliver care, but many fail to keep users engaged long enough to be useful. Surveys can capture what users *say* about their experience, but understanding how they actually use an app — which buttons they press, which screens they linger on, where they get stuck — offers complementary insight. This detailed usage data, called paradata, is rarely collected or analyzed in digital health. In this study, we analyzed paradata from the mLab App, a mobile-friendly web application that guides users through at-home HIV self-testing. The app was used by 210 young men who have sex with men across New York City and Chicago as part of a clinical trial. We found that users engaged with the app in varied ways: many sessions were brief, a surprisingly large number of users accessed the app from a computer rather than a phone, and the test-photo upload step often required retries to meet image-quality checks. Educational features and personalization options were rarely used. These insights show how paradata can reveal where digital health tools succeed, where users encounter friction, and where they can be improved — lessons that apply well beyond HIV testing.

## Introduction

The rapid integration of digital tools in healthcare over the past two decades has transformed patient care, providing new avenues for diagnosis, monitoring, and treatment [1–4]. This surge in digital solutions has necessitated the development of sophisticated analytical tools to ensure these applications are user-friendly and effective [5,6]. Paradata, data that details the events and processes in which users engage with an application (i.e., screen loads, button clicks, scroll events), which encompasses detailed insights about user interactions within an app, offers a promising avenue to understand and improve tool utilization across various platforms [7–9]. Although paradata has been employed in small pilot projects by our group and a few others [10–12], its substantial potential remains largely untapped in digital health, underscoring a gap in the utilization of digital tools in healthcare [13].

While paradata is extensively utilized in the commercial, educational, gaming, and media sectors – such as e-commerce to refine user experiences and optimize business outcomes [14] or monitoring of remote students’ test taking [15,16] – its application within digital health remains nascent [17]. The challenge is even more pronounced in underserved populations at risk for HIV infection [7–9]. This discrepancy underscores the need for data-driven enhancements in healthcare, similar to those in the commercial sector [18]. Beyond patient-facing optimization, paradata can also be used as a research instrument where session- and event-level logs help reduce response variability by pinpointing where instructions or terminology need clarification, improve time efficiency by revealing navigation bottlenecks, identify potential sources of measurement or selection bias (including device-/browser-specific friction), and increase ease of use for participants and study staff. By detailing our approach to paradata analysis, this research seeks to demonstrate how paradata can be effectively gathered and leveraged to enhance digital health applications, particularly focusing on HIV diagnostic testing. Although commercial platforms have established paradata practices, health-specific implementations require unique considerations, including clinical decision support workflows, regulated diagnostic steps, and safety-critical interpretation of results. To date, there are no published exemplars demonstrating how to operationalize event-level paradata for regulated health diagnostics, nor methods for detecting friction in clinical task sequences, discordance awareness, and session-level adherence to testing windows. This study contributes a portable measurement framework for structured paradata capture in health applications.

HIV self-testing (HIVST) is one area where paradata may add value in healthcare, as it has emerged as an important tool to expand access to diagnosis among difficult to reach populations [19,20]. Digital platforms like the mLab App support this effort by guiding users through self-testing and providing automated result interpretation and follow-up resources. These tools are increasingly relevant to HIV prevention, including linkage to care and initiation of pre-exposure prophylaxis (PrEP), where timely and accessible testing is critical [21,22].

**Fig 1:**
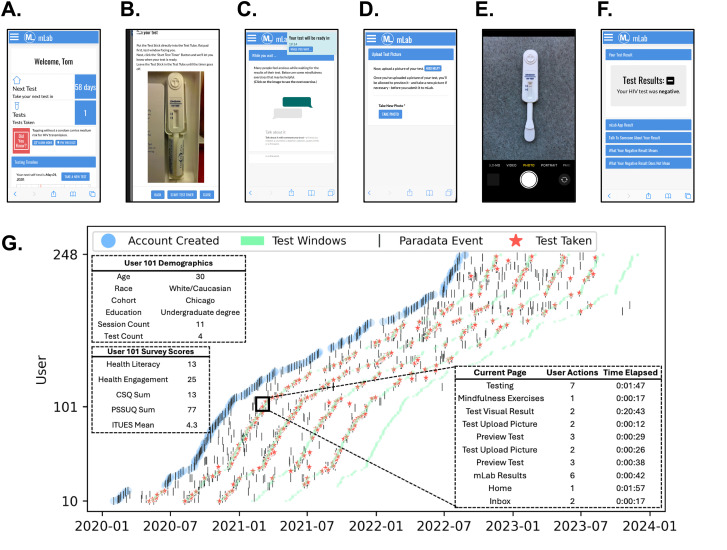
The mLab App A) home screen, B) test instructions, C) wellness exercises during testing, D) test photograph upload instructions, E) participant-captured photograph of test, and F) test results. **G)** Timeline of paradata collection throughout the duration of study. A paradata event is a logged app interaction (e.g., page view, button click, scroll, authentication, test start, image upload, result view, logout) recorded with timestamp, session ID, and anonymized user ID. In the timeline panel, each mark represents a paradata event for a single participant; the horizontal axis shows Time Elapsed (minutes since session start), and rows (e.g., “User 101”) denote anonymized participant IDs. Insets highlight a single testing session from a participant, along with the demographic information and survey responses from that participant. Abbreviations in inset: CSQ (Client Satisfaction Questionnaire), PSSUQ (Post Study System Usability Questionnaire), ITUES ([Health] Information Technology Usability Evaluation Scale).

Our study utilizes the mLab App (Figs 1A–1F and S1), a mobile-friendly web application developed to promote HIV testing within a large, multi-site clinical trial conducted across New York City and Chicago. The app collected extensive paradata – including authentication times, time spent on individual pages, navigation paths, and other user interactions – providing a rich dataset for analyzing user engagement (Fig 1G). This manuscript presents findings from the mLab App, including patterns of user interaction and feature usage, and introduces methodological approaches for analyzing paradata. By examining in-app behaviors, we generate insights that can inform improvements to app functionality and user experience, demonstrating the broader potential of paradata to optimize digital health tools.

## Methods

### Study design and participants

This study was conducted as part of a large, multi-site three-arm randomized clinical trial spanning nearly four years, with sites located in New York City and Chicago [23]. The mLab App and study protocol, as well as clinical trial efficacy and outcomes have been described in detail elsewhere [23,24]. The primary aim of the trial was to assess the impact of the mLab App on HIV testing uptake rates, comparing these rates with a groups that received mailed home tests but did not use the app and a group that received standard of care education on testing and PrEP. A secondary aim was to evaluate the usage of pre-exposure prophylaxis (PrEP) for HIV prevention among participants. The longitudinal trial enabled the collection of extensive paradata through the app, which was analyzed to better understand user interactions and system performance over time. Key eligibility criteria included age 18–29; assigned male sex at birth (including TGW); English literacy; smartphone ownership; CDC risk for HIV; HIV-negative or unknown status (verified at enrollment); no HIV test in the prior 6 months; not currently on PrEP; and understanding of OraQuick limitations. Participants were excluded for known HIV diagnosis or investigator safety concerns.

### Data collection and procedures

Paradata collection functionality within the mLab app was implemented entirely in JavaScript, designed to interact with our custom-built PHP API, similar to that described by Khasawneh et al. [25] In this manuscript, a “paradata event” is a discrete, app-generated log of user interaction recorded with timestamp, session ID, anonymized user ID, and event type (e.g., page view, button click, authentication, test start, image upload, logout). A “session” is a contiguous period of activity beginning at authentication and ending at logout or inactivity timeout. This aspect of the app operated asynchronously, ensuring that data collection ran smoothly in the background without disrupting the user experience. Relying entirely on asynchronous logging, it allowed study participants to engage with the app without any noticeable delay or interruption. All paradata collected were stored using anonymized, coded user IDs without any direct personal identifiers. Data were stored within a secure cloud environment. Participants were informed during the consent process that their interactions within the app would be logged for research purposes.

Throughout the development of the mLab app, multiple rounds of usability testing were conducted to refine and enhance the user interface [26]. These tests were specifically designed to gather feedback from our target demographic, ensuring that the app’s design and functionalities aligned with their needs and preferences. These iterative evaluations were performed to improve the app’s overall usability but also ensured that the interface was intuitive and effective for the intended users, enhancing engagement and the accuracy of data collection.

Participants were instructed to use the mLab App in conjunction with provided HIVSTs. After authenticating, users could initiate a guided walkthrough of the self-testing process, which included step-by-step visual instructions, a built-in timer aligned with the 20-minute test development time, and optional mindfulness exercises during the waiting period. After completing the test, users were prompted to photograph the test device, which was analyzed by the mLab App’s image processing algorithm to provide an experimental result. Participants recorded their visual interpretation as a categorical outcome –negative, positive, or invalid – following the test manufacturer’s instructions. The app’s computer-vision computes a continuous score internally but displays a categorical interpretation using a laboratory-determined threshold (negative/positive) and flags invalid when quality checks (e.g., missing control line, severe blur/lighting) fail; only the categorical interpretation is shown to participants. Users could then review both their self-reported (visual) result and the app’s interpretation, and personalized content based on their testing results. Additional app features included access to educational content and HIV prevention resources, including information on PrEP.

### Session and device identification

We model the data using a hierarchical structure with three levels: participants, sessions, and events. Participants are represented by anonymized, coded user IDs. A session is a contiguous period of activity beginning at authentication and ending at logout or inactivity timeout; each session has a unique session ID and is linked to its participant via the anonymized user ID. Events are discrete logged interactions (e.g., page view, button click, authentication, test start, image upload, result view, logout) recorded with timestamp, session ID, and anonymized user ID. Because a participant may complete more than one session, those sessions are longitudinally linked across the study period for that participant.

At each level we record: participant level—anonymized user ID, trial arm (mLab App arm), and enrollment/authentication status; session level—session ID, start/stop timestamps, total duration, counts of page views and actions, and whether a test was initiated/completed during the session; event level—timestamp, event type (e.g., page view, button click, authentication, test start, image upload, result view, logout), page/screen identifier, session ID, and anonymized user ID. For each session we also classify platform (mobile vs. desktop) and record operating system and browser by parsing the User-Agent string and viewport information at session start; these attributes are stored with the session record. Because participants may have multiple sessions, we index sessions in within-participant sequence order enabling per-participant timelines and aggregate trend analyses by session sequence and relative time.

### Data visualization and statistical analysis

Custom Python scripts utilizing pandas (v2.0.1), matplotlib (v3.7.1), seaborn (v0.12.2), scikit-learn (v1.2.2), and SciPy (v1.10.1) were used to analyze paradata from the mLab App. We summarized usage with four outcomes: accessed pages (the page/screen identifier logged for each event), navigation paths (within-session ordered sequences of page/screen identifiers), action responses (user-initiated events—e.g., authentication, button clicks, test initiation/completion, logout—summarized per session and per participant), and session metrics (duration, counts of page views/actions, and whether testing occurred). For analyses, we constructed a filtered dataset from raw logs restricted to authenticated participants in the mLab App arm and the prespecified analysis window, excluding staff/developer/test events. From this we built derived datasets: (1) session-level summaries, (2) participant-level summaries, and (3) within-session transition matrices of page movements. Descriptive statistics were computed for filtered and derived datasets (counts, medians, interquartile ranges, means, standard deviations, and extrema). Visualizations (matplotlib/seaborn) included histograms, cumulative distributions, and bar charts of usage frequencies, navigation paths, and session metrics; we also summarized temporal engagement at two scales—within-session (elapsed minutes since session start) and across-session (within-participant session sequence and days since first authentication). Primary usage metrics were (i) total in-app time per participant (sum of session durations), (ii) session count and session duration, and (iii) paradata event count per participant.

### Ethical considerations

The study was reviewed and approved by the Institutional Review Boards at Columbia University, Lurie Children’s Hospital in Chicago, and Vanderbilt University. The use of the mLab App was reviewed by the US Food & Drug Administration and assigned an Investigational Device Exemption (FDA IDE 18348). All data collection procedures, including the collection of paradata, were approved as part of the ethical review to ensure participant privacy and confidentiality.

## Results

### Paradata cohort

Over the duration of the parent randomized trial, 525 participants were enrolled across three arms; 210 participants were enrolled into the mLab App arm of the study. The raw paradata was filtered to remove events from study team and development team members, as well as paradata generated before and during the process of – but prior to – authentication (which was collected for security purposes). A total of 19,458 paradata events were generated by authenticated participants during the study period. These filters yield the dataset used for all analyses. A timeline of the user count (participant enrollment into the app arm of the study) and the count of in-app participant-generated paradata is shown in S2 Fig.

Participants were young men who have sex with men (YMSM), 18–29 years (mean 24.3, SD 3.2), recruited in New York City and Chicago, with racial/ethnic diversity (approx. 58.3% White, 11.8% Asian, 10.4% Black/African American, 10.0% multiracial, 5.7% “something else,” 0.9% American Indian/Alaskan Native) and ~34.6% Hispanic/Latino; education spanned less than high school through graduate degrees. Full participant demographics are described in detail elsewhere [24,27].

### Overall app- and device-usage distributions

**Fig 2:**
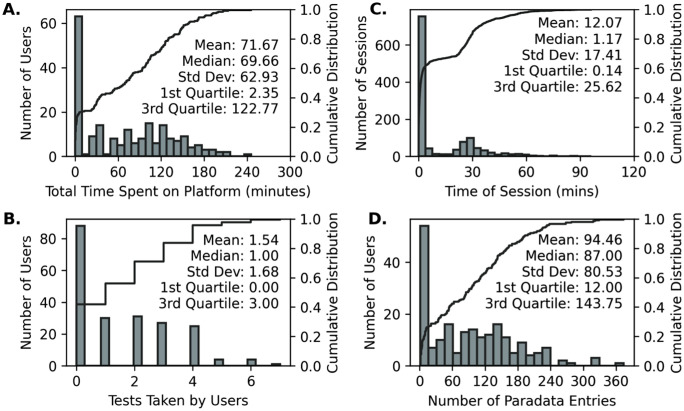
Distributions and cumulative distribution functions of A. the total time spent in the app, **B.** number of tests taken by users, **C.** durations for app sessions, **D.** number of paradata entries generated by each user.

Distributions of total in-app time, number of tests taken per user, session duration, and paradata events are summarized in Fig 2. Median total time was 69.66 min (IQR, interquartile range, 2.35–122.77); 30% of users (n = 63) spent less than 10 minutes in the app. Tests taken per participant had a median of 1 (IQR 0–3). Session duration was skewed (median 1.17 min, IQR 0.14–25.62; mean 12.07 min). Participants generated a median of 87 paradata events (IQR 12–143.75).

Temporal distributions of session start time and day of the week, as well as test start time and day of the week, were heterogeneous – including mostly equal device type usage (S3 Fig). App usage, measured by the start of a unique session, was mostly evenly distributed across weekdays, with a substantial drop-off in the number of sessions started on weekends. HIV tests were most frequently performed on Wednesday and Monday, and least frequently performed on Sunday. Sessions and tests both abruptly increased around 9am, peaking in the middle of the day; the number of sessions continued decreasing throughout the evening, while the number of tests performed was relatively consistent through midnight.

**Fig 3:**
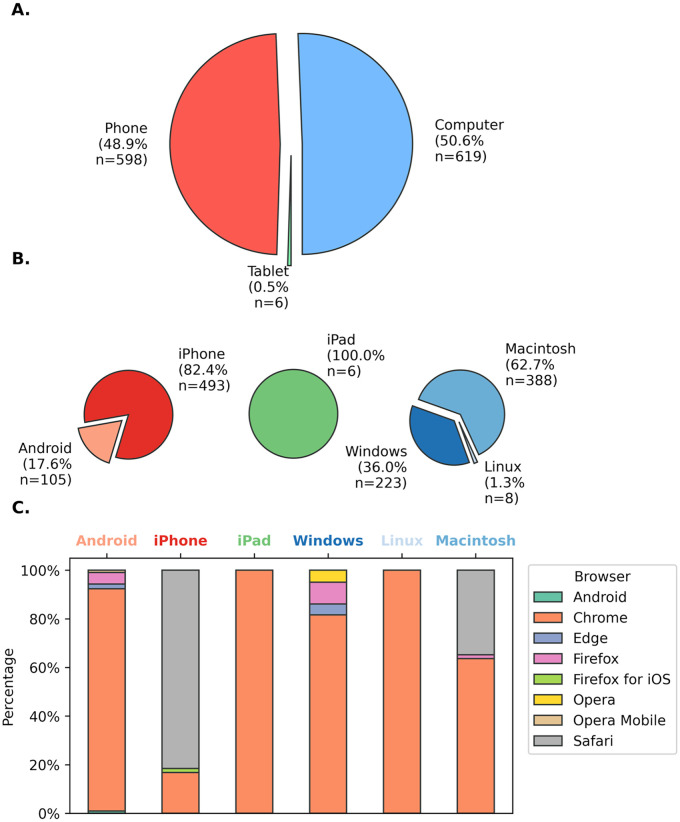
Application usage (measured by percentage of sessions) by device type, device model, operating system, and browser.

We observed more desktop sessions than mobile sessions, and very few tablet sessions (Fig 3). Within mobile sessions, iPhones were much more common than other devices; similarly, Apple computers were the most frequently used to access the mLab App. On iPhones, the most commonly used browser was Safari; on all other devices, Google Chrome was the most common. Looking at individual user behavior, we found that most users accessed mLab through only one device throughout their participation in the study (S4 Fig). However, 74 users used more than one device throughout the study to visit mLab.

### Feature usage

**Fig 4:**
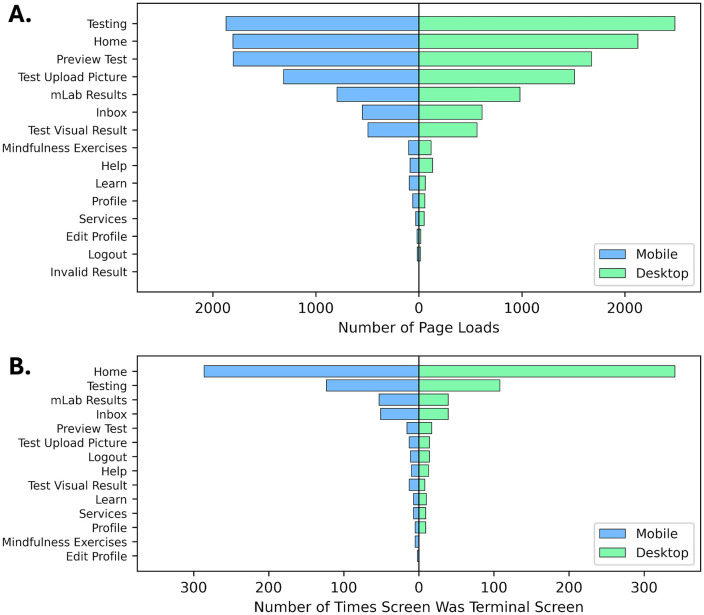
(A) count of page loads by specific page, and (B) the number of times each loaded screen was the final screen a user viewed during their mLab session, grouped by device type.

Paradata was analyzed to determine the frequency of usage of each screen and feature (Fig 4), as well as to investigate usage patterns (S5 Fig).

**Fig 5:**
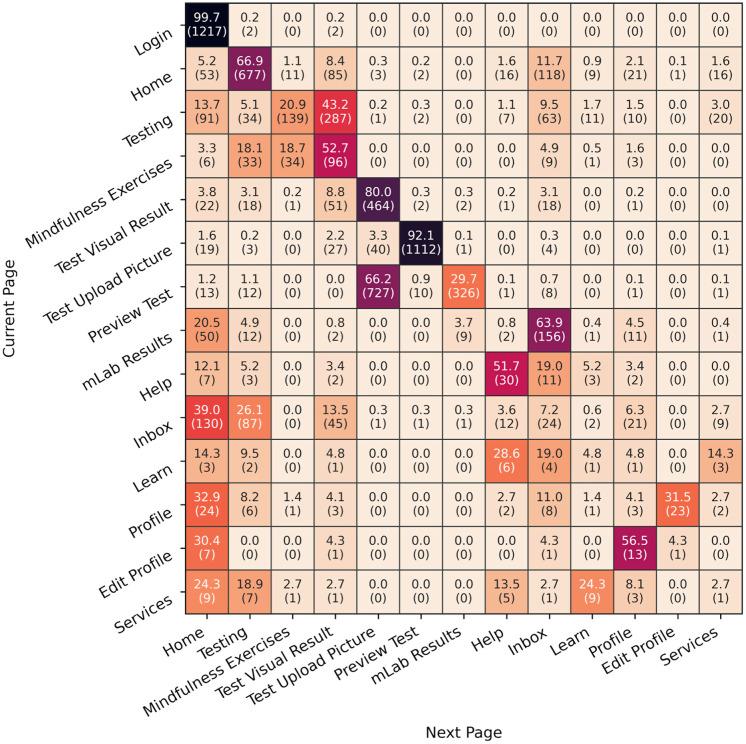
A heat map of navigation pathways, from the current page (y-axis) to the next page (x-axis). The navigation pathway is scaled to 100% along the current-page dimension (actual counts are shown in parentheses). For any given row, each column shows the percentage of times that screen was the next screen, from the row’s current page, in a user’s navigation history.

The Testing screen was the most frequently loaded, even more so than the Home screen where users are directed there after successful authentication. There are different steps in the testing workflow that redirect users back to the testing page (i.e., after initiating a test, after analyzing a test). Aside from Home, the next most frequently loaded pages were also related to testing (i.e., Preview test, Test Upload Picture, mLab Results). As the image processing algorithm checked photo quality and recommended retakes if quality was low or the test could not be detected, depending on how many photos the user had to take before one was able to be analyzed, the “Preview Test” and “Test Upload Picture” screens could have been loaded multiple times in the same testing workflow before navigation to Test Visual Result. In design, the intended path is largely linear (Test Start–Upload Picture—Preview—Result); the row-normalized next-step probabilities in Fig 5 allow a visual check of how closely observed flows align with that goal path. We observed more cyclic movement between Upload and Preview than anticipated, consistent with photo-quality retries. This cyclic nature is quantified in Fig 5, and further visualized in the strong bands in the Sankey diagram between these two screens (S5 Fig, an aggregate Sankey diagram across all participants and all sessions with screen-to-screen transitions pooled). After Test Visual Result, users typically move to Test Upload Picture (80%, 464 of 580 next screen loads). From Test Upload Picture, users most frequently move to Preview Test (92.1%, 1112 of 1,207 next screen loads). The next pages from Preview Test are most frequently Test Upload Picture (66.2%, 727 of 1,099 next screen loads), demonstrating the sometimes-cyclic nature of capturing an ideal photograph for image analysis, and mLab Results (29.7%, 326 of 1,099 next screen loads). Mobile- and desktop-specific transition heatmaps were broadly similar, with the Upload-Preview loop present on both platforms (S6 Fig). Among sessions reaching a result (n = 314), the median time from session start to receiving a result was 24.97 minutes on mobile (mean 25.28) and 29.90 on desktop (mean 33.23), a modest 4.93-minute median difference. After receiving their test results on the mLab Results screens, most users went to check their new notifications in their Inbox (63.9%, 156 of 244 next screen loads).

Screens that received little traffic throughout the duration of the study included educational materials (i.e., Help, Learn, Services), as well as screens that allow more user customization (i.e., Profile, Edit Profile). Another under-utilized feature, the Logout screen (loaded when a user pressed “Logout” within the drawer navigation menu), was the terminal screen in a session with only 25 logouts, out of 1223 sessions. Clearly, most sessions ended through the session timeout mechanism, and the most common terminal screens (i.e., screens where the last paradata entry in a session occurred) were Home, Testing, mLab Results, and Inbox. Substantial differences in terminal screen across device type were not observed (Fig 4).

Beyond page loads, paradata can also be used to understand granular user actions on each page. In our study, we dealt with an investigational diagnostic tool that presented users the result of record (the participant’s visual inspection of their test) in addition to the experimental result (the mLab App’s image processing algorithm result). As such, when these results disagreed with one another, it was important to understand whether users were aware of the discordant results so they could be counseled on the correct result (a complete analysis of discordant results is the subject of other work [28]). To this end, we closely observed actions on the mLab Result screen and the user’s inbox, two locations where users could see results of their recent tests (S7 Fig). We found that most users (75%, n = 236) did actively click to expand the section “mLab App Result” to see the results of the experimental image processing algorithm, even after being shown a disclaimer that their result of record was the visual result (the visual result was viewed 91%, n = 286, of the time). Educational information describing what a negative test does and does not mean were viewed 48% (n = 152) and 44% (n = 138) of the time, respectively. The “Talk to someone about your results” button was clicked 45% (n = 142) of the time, indicating that immediately after a test is performed there is a need for counseling. Inspection of whether or not users opened messages in their mLab App inbox showed that 48% (n = 198 of 413 messages) of users opened messages that had the subject “New test” and conveyed a summary of their most recent test results. A similar percentage of “Welcome to mLab” messages were opened (47%, n = 98 of 210 messages). While usage of the Help screen was relatively low compared to other features, 71% (n = 22 of 31 messages) of messages with the subject “Help is on the way!”, sent after a help request was made, were opened.

The “Help” functionality within the mLab App was designed as a one-way communication system, allowing users to alert the study team if assistance was needed. Users selected a predefined help topic, and follow-up was conducted directly by study personnel outside of the app. In-app two-way chat functionality was considered during the design process but was not implemented in order to limit the burden on study staff and avoid open-ended communications. While response time was not directly recorded within the app, internal study logs documented and timestamped all incoming requests and responses. Most participant help requests were addressed within one business day.

While there was educational content in the mLab App, participant usage of these features was limited. Despite the substantial volume of educational content on the home screen, visits to dedicated educational screens like “Learn” and “Services” were low; no notable difference on feature usage was seen on different device types (Fig 4). Differences in page loads, Throughout the duration of the study, an educational “fact” (a piece of knowledge related to HIV, PrEP, PEP, and mLab) in lay terms, from a set of 44 curated by the study team, was shown on the home screen 1654 times. While users had the ability to “pin” a fact to their profile and visit it later, this feature was used only 8 times by 7 different users.

## Discussion

In this study, we used paradata to examine how users engaged with the mLab App, a digital HIV self-testing platform. We identified five key insights: (1) overall app usage patterns showed a wide distribution of app engagement, including a substantial number of brief sessions and minimally engaged users, alongside a contingent of more engaged users; (2) contrary to expectations, most users appeared to access the app from desktop or laptop computers rather than smartphones; (3) particular app features (e.g., educational content and external services) were underutilized; (4) users frequently re-engaged with specific parts of the diagnostic testing workflow, especially test image upload, indicating an iterative or cyclic workflow; and (5) session closure behaviors varied, with some users abandoning testing workflows and few users using the logout feature. These findings have implications for app design, user onboarding, diagnostic flow optimization, and security practices, and suggest several areas for future refinement in digital health tools that support at-home HIV testing.

The expansion of digital health applications, especially during and after the COVID-19 pandemic, has raised new questions about sustainability, user engagement, and long-term effectiveness [1,29]. Despite enthusiasm and investment, many digital tools suffer from low uptake, high abandonment, and limited scalability [30,31]. This phenomenon, sometimes described as “app fatigue” or “pilotitis,” is often driven by a poor understanding of how users engage with the technology [32–34]. Paradata—passively collected in-app usage data—offers a promising avenue to close this gap by revealing user behaviors that can inform app refinement, usability improvements, and implementation strategies. Beyond descriptive usage statistics, our analyses demonstrate how event-level paradata enable detection of task-specific workflow friction, measurement of post-discordant behavior, and quantification of adherence to testing windows. These analytic strategies offer a methodological template applicable to other regulated digital health interventions. We provide open data and code to enable replication and adaptation for future mHealth research.

### Overall app- and device-usage distributions

Across the entire participant population, application usage metrics offer a glimpse into in-app user behavior. For each of the metrics (total time spent on platform, time of session, number of paradata entries), we see that there is a notable subset of the data indicating only brief usage. It is clear that a minority of users had limited usage – visible through the total time spent on the platform and the number of paradata entries. The median session time is only 1.17 minutes, and more than 700 sessions were less than 4 minutes – for comparison, after adding the swab from the OraQuick test to the running buffer, the test itself takes 20 minutes to complete. Engagement shows two predominant patterns: brief or zero-interaction sessions (e.g., quick check-ins/navigation less than 10 minutes in duration) and longer sessions consistent with end-to-end self-testing. Paradata—combining session duration with event sequences—helps differentiate these task types and reveals substantial dispersion, as noted by IQRs. A cautious interpretation is that longer sessions reflect testing workflows, whereas shorter sessions may reflect exploratory or reminder use. This would align with the Uses and Gratifications Theory, suggesting that mLab App sessions were mostly intentional with specific goals [35]. Understanding temporal app usage patterns holds significant implications not only for the delivery of interventions but also for the development and maintenance of the applications themselves, where they can be used to gauge cloud infrastructure specifications. There was a drop off in session usage over the weekends, however this pattern was not necessarily visible when looking at tests only. Most sessions and tests occurred near the middle of the day, again an important note for managing the scaling of cloud infrastructure.

Surprisingly, for a mobile health application, we found that most users actually used the application on a computer (we did not differentiate between desktops and laptops). As a mobile-friendly web application, the mLab App was designed to be device agnostic, but we had anticipated a majority of sessions would occur on mobile devices. Enrollment criteria included the requirement of smartphone ownership, but participants were not required to use only their mobile phone, indicating an interesting preference amongst our study population. From previous work with a mobile-friendly web application for contact tracing during the COVID-19 pandemic, we have seen user preference for native applications over web applications – particularly for non-stationary tasks that are to be accomplished in multiple locations [11,36].

Considering the sensitive nature of HIV self-testing, many users may prefer the privacy and comfort of completing this task at home, where they have ready access to their desktops or laptops, making these devices a reasonable option. Our users did seem to use non-phone devices with an unexpected frequency, however it is unclear how generalizable this finding may be to other populations and more exploration is necessary. Still, this unexpected finding, and the appearance of edge cases in devices, hardware, and browsers speak to the need for thorough feature and compatibility testing during development in digital health applications. While there may be interesting socioeconomic analyses of our users’ choice in technology [37], analysis of demographics of device or brand selection was beyond the scope of this study.

The device types, device manufacturers, operating systems, browser, and browser versions described in this work are all derived from the user agent that is sent with every HTTP request made to the server. User agents share details of the sender’s device and software so the server can properly format optimal and compatible content for return [38]. Analyzing the user agent string is an industry standard method for understanding device and browser usage on websites, allowing developers and marketers to optimize user experience and tailor content delivery. Nonetheless, user agents can be altered through privacy software and settings (i.e., VPNs, browser security extensions) and mobile browsers mimicking desktop browsers to request desktop versions of websites. While we used the first user agent from each session for results in Fig 3, we did observe 5 sessions that changed user agents within a single session, indicating some alteration of the user agent on the client side. Furthermore, recent tablet operating systems from Apple (iPadOS 13+) alter user agents to request desktop versions of websites, while Windows Surface tablets run full versions of desktop web browsers on a desktop operating system. In these instances, the user agents would not be able to accurately identify these devices and browsers. Thus, it is possible that the actual number of desktop devices could have been lower, and phones and tablets could have had a higher proportion of usage than reported in Fig 3. Since all features and functionality of mLab App were available on both mobile and desktop devices, and high accuracy device fingerprinting [39] was not a necessity for our study, we believe device and browser selection was a user preference and likely had little outcome on application usage. In situations where correct device identification is critical to web application use, alternative approaches like querying screen size, resolution, device orientation, and touch capabilities can supplement user agent analysis and improve device identification accuracy.

### Feature usage

It is useful to recognize that features identified as desirable during pre-launch focus groups may not always translate into frequent use. In mLab App, we identified several features that received little traffic: educational resources, external services, and personalization options. From a development perspective, identifying these underutilized features allows focus to be applied to other needs – or to understand why these features are not receiving the anticipated usage.

Paradata allows for identification of potential in-app navigation barriers encountered by users. We identified circular navigation patterns, particularly in the testing workflow, as users iterated through uploading a photograph of their diagnostic test with a 66.2% retake rate (retake rate was 67.6% on mobile; 64.6% on desktop). This is partially related to strict photo quality assurance criteria in place as part of mLab’s use as an experimental diagnostic aid – it is safer to ask users to retake their photograph than to potentially analyze it incorrectly because it did not meet the necessary image processing requirements. As HIV status is a particularly critical piece of protected medical information [40], there is a delicate balance between diagnostic accuracy and user-experience that must be obtained for any app of this type. In this case, and throughout the app as a whole, user flow controls must be carefully crafted and monitored, especially in web applications where the browser’s back button provides an alternative navigation mechanism, which could lead to unintended consequences if not properly managed. Row-normalized transition matrices (as in Fig 5) are a standard exploratory paradata tool for visualizing within-session navigation. In this framing, “good design” concentrates next-step probability along the intended path (e.g., Upload—Preview—Result) with minimal backtracks or dead-ends; the pronounced Upload—Preview cycling highlights a last-mile friction point for future redesign.

In sensitive settings like healthcare, leveraging paradata allows study personnel to confirm whether a user has seen specific essential information. Our study utilized paradata to determine if a user was aware of discordant results between the mLab App and their self-reported visual result. On different screens where results were available, users were required to take an explicit action (i.e., actively click on a collapsible card with their mLab results, load their test history table, or click a message in their mLab inbox) to see the mLab algorithm’s experimental results. Thus, these interactions could be recorded, and they were critical for assessing whether users were likely aware of discordant results and shaping follow-up conversations with participants.

Similarly, understanding how users end their sessions has vital implications for application security. While the mLab App had a session timeout automatically set for security purposes, the number of times the “Logout” button in the navigation menu was actively pressed was rare. This reinforces that securely closing sessions should not be left solely to a user action, and best practice would be to also utilize an inactivity auto-timeout. Through paradata analysis, we were also able to understand that users sometimes ended their session in the midst of their testing workflow, leaving a testing session incomplete. Further examination of user flows revealed that users took advantage of the application’s “persistence”, where testing sessions could be resumed assuming the user was still within the acceptable time for analysis of their HIVST. Effectively, this allowed users to start and finish a test in different sessions. While this was infrequently used, it could impact aggregate metrics like total session count and time per session. Future iterations of the application are being tested with architectural modifications to track such user flows as a single contiguous session.

### Translating insights into app improvements

Together, these findings highlight key areas for improving the usability and effectiveness of digital self-testing companion tools. In response to the high rate of brief sessions and image retakes, future versions of the mLab App are being adapted to include improved onboarding instructions, and a more forgiving image processing algorithm. Underutilized features such as educational content and HIV-related resources are being reconsidered for redesign or repositioning within the interface. Moreover, the unexpected preference for desktop use has prompted both a renewed emphasis on cross-platform compatibility and design consistency across device types, as well as consideration of porting the mLab App to a native mobile application. By directly translating paradata insights into architectural and design modifications, we aim to reduce friction in the testing process, support sustained engagement, and ultimately increase the feasibility of routine HIV self-testing in diverse populations.

Companion analyses [27,28] of the same trial show several ways that paradata can be paired with outcomes to inform redesign and evaluation: (i) Prompt effectiveness & adherence to testing schedules—74.2% of all HIV self-tests occurred in the hard testing window following reminders, suggesting a direct path to measuring “tests-within-window” as an outcome for adaptive nudging strategies; (ii) Last-mile friction after the timer—three post-timer screens (Preview Test, Upload Picture, Visual Result) account for 60.6% of incomplete testing sessions, enabling pre/post A/B tests that target these steps and read out completion-of-result-submission and time-to-result; (iii) Behavior after discordant results—users who experienced an automated/visual discordance showed a short-term drop in session activity without long-term reduction in testing, motivating just-in-time messaging with outcomes such as recovery time to next session/test and linkage to confirmatory care; (iv) Cross-device continuity—observed phone-desktop handoffs across sequential sessions support measuring whether cross-device affordances improve completion rate and time-to-result. In parallel, paradata-defined engagement clusters (Minimal/Moderate/Power) correlate with perceived usability (Health-ITUES) and health engagement, suggesting a personalization route where cluster-specific onboarding or prompts are evaluated on test frequency, time-to-test, and sustained use.

### Limitations

This analysis uses paradata from a single web application deployed within a randomized clinical trial and is therefore context-specific. Generalizability is limited to English-reading young men who have sex with men (YMSM), aged 18–29, recruited in two urban U.S. settings (New York City and Chicago); although the App arm was racially and ethnically diverse, findings do not extend to non-English speakers, women, older adults, or rural populations. Because participants could complete multiple sessions, observations are not independent; we therefore emphasize per-participant summaries, non-parametric distributional statistics (medians/IQRs), and descriptive flow analyses rather than inferential models that assume independence. Paradata capture what screens were visited and when, but not user intent; some behaviors (e.g., reading off-app materials, care-seeking outside the app) are not observable. Finally, this manuscript focuses on usage patterns and measurement methods and does not link paradata to clinical outcomes (e.g., test completion, positivity, linkage to care) or make inter-arm efficacy claims; those results are reported elsewhere.

Importantly, the analytic approach presented here is not specific to mLab. Any digital health platform that contains safety-critical workflow steps (self-testing, medication adherence, automated decision support) can adapt these event structures and analytic procedures. Our goal is not merely to report usage patterns, but to provide a reusable paradata measurement framework for digital health evaluation.

## Conclusions

Paradata is easy-to-collect in the background of an application without disrupting the user experience. While it can offer valuable insights into usage patterns, paradata has been underutilized in digital health applications. We collected paradata during a multi-site, randomized clinical trial of an FDA-IDE approved mobile health web application designed to assist with HIV self-testing. In this report, we described usage patterns, both at a high-level and at an individual level, including page visits, time spent, and specific feature usage. These findings align with our study aim to assess how detailed interaction logs can inform design improvements, support user-centered development, and provide a deeper understanding of mHealth application usage. When combined with participant demographics and survey responses analyzed in complementary work [27], along with our other efforts focused on an in-depth analysis of the paradata associated with self-testing patterns within the app [28], we hope to inspire further research that explores innovative ways to leverage this underutilized resource in healthcare and beyond. This investigation could lead to predictive modeling to understand user needs and deliver highly personalized interventions [41]. By sharing our findings and diverse methods for analyzing paradata, we aim to provide a blueprint that others can adopt and modify to enhance detailed usage analysis of their applications. To facilitate further research, we have published a cleaned version of our raw paradata, a data dictionary, and the code used in this analysis, available alongside this manuscript. We hope this will foster further advancements in the field of digital health, particularly in user-centered design, continuous evaluation, and iterative development and delivery.

## Supporting information

S1 Fig Representative screenshots of the desktop layout of the mLab App. A. Landing page. **B.** Preview test page.
(DOCX)

S2 Fig User enrollment and total paradata count throughout the duration of the study. (DOCX)

S3 Fig Temporal counts of sessions and tests, stacked by device type. **A.** Number of sessions by day of the week. **B.** Number of sessions by hour of the day. **C.** Number of tests by day of the week. **D**. Number of tests by hour of the day.
(DOCX)

S4 Fig Number of unique devices each user used, per the browser agent of the first paradata entry of each session. (DOCX)

S5 Fig Sankey diagram detailing the flow of traffic from screen to screen within the mLab application. (DOCX)

S6 Fig Heat maps of navigation pathways, from the current page (y-axis) to the next page (x-axis), isolated by (A) mobile and (B) desktop sessions. The navigation pathway is scaled to 100% along the current-page dimension (actual counts are shown in parentheses). For any given row, each column shows the percentage of times that screen was the next screen, from the row’s current page, in a user’s navigation history.
(DOCX)

S7 Fig A. The percentage of sessions that reached the results page where a user performed a specific action on B. the Results page. C. The percentage of alerts that were read by participants in D. their mLab inbox. (DOCX)

## References

[1] Manteghinejad A, Javanmard SH. Challenges and opportunities of digital health in a post-COVID19 world. J Res Med Sci. 2021;26:11. doi: 10.4103/jrms.JRMS_1255_20 34084190 PMC8103966

[2] Abernethy A, Adams L, Barrett M, Bechtel C, Brennan P, Butte A, et al. The promise of digital health: then, now, and the future. NAM Perspect. 2022;2022:10.31478/202206e. doi: 10.31478/202206e 36177208 PMC9499383

[3] Mathews SC, McShea MJ, Hanley CL, Ravitz A, Labrique AB, Cohen AB. Digital health: a path to validation. NPJ Digit Med. 2019;2:38. doi: 10.1038/s41746-019-0111-3 31304384 PMC6550273

[4] Kao CK, Liebovitz DM. Consumer mobile health apps: current state, barriers, and future directions. PM&R. 2017;9:S106–15. 10.1016/j.pmrj.2017.02.018 28527495

[5] Huckvale K, Wang CJ, Majeed A, Car J. Digital health at fifteen: more human (more needed). BMC Med. 2019;17(1):62. doi: 10.1186/s12916-019-1302-0 30879466 PMC6421699

[6] Pham Q, Graham G, Carrion C, Morita PP, Seto E, Stinson JN, et al. A library of analytic indicators to evaluate effective engagement with consumer mHealth apps for chronic conditions: scoping review. JMIR Mhealth Uhealth. 2019;7(1):e11941. doi: 10.2196/11941 30664463 PMC6356188

[7] Bauermeister JA, Golinkoff JM, Muessig KE, Horvath KJ, Hightow-Weidman LB. Addressing engagement in technology-based behavioural HIV interventions through paradata metrics. Curr Opin HIV AIDS. 2017;12(5):442–6. doi: 10.1097/COH.0000000000000396 28617711 PMC5637536

[8] Choi SK, Muessig KE, Hightow-Weidman LB, Bauermeister JA. Paradata: measuring engagement in digital HIV interventions for sexual and gender minorities. Curr HIV/AIDS Rep. 2023;20(6):487–501. doi: 10.1007/s11904-023-00679-5 37930613

[9] Hightow-Weidman LB, Bauermeister JA. Engagement in mHealth behavioral interventions for HIV prevention and care: making sense of the metrics. Mhealth. 2020;6:7. doi: 10.21037/mhealth.2019.10.01 32190618 PMC7063263

[10] Chen AT, Chang JH, Hallinan S, Mohr DC. Mapping user trajectories to examine behavior and outcomes in digital health intervention data. 2019 IEEE Workshop on Visual Analytics in Healthcare (VAHC); 2019. p. 1–8. doi: 10.1109/VAHC47919.2019.8945038

[11] Scherr TF, DeSousa JM, Moore CP, Hardcastle A, Wright DW. App use and usability of a barcode-based digital platform to augment COVID-19 contact tracing: postpilot survey and paradata analysis. JMIR Public Health Surveill. 2021;7(3):e25859. doi: 10.2196/25859 33630745 PMC8006896

[12] Chen AT, Wu S, Tomasino KN, Lattie EG, Mohr DC. A multi-faceted approach to characterizing user behavior and experience in a digital mental health intervention. J Biomed Inform. 2019;94:103187. doi: 10.1016/j.jbi.2019.103187 31026595 PMC6662914

[13] Guo C, Ashrafian H, Ghafur S, Fontana G, Gardner C, Prime M. Challenges for the evaluation of digital health solutions-A call for innovative evidence generation approaches. NPJ Digit Med. 2020;3:110. doi: 10.1038/s41746-020-00314-2 32904379 PMC7453198

[14] Peters R. Growth hacking techniques, disruptive technology - how 40 companies made it big. World Ideas Limited; 2014.

[15] Diedenhofen B, Musch J. PageFocus: using paradata to detect and prevent cheating on online achievement tests. Behav Res Methods. 2017;49(4):1444–59. doi: 10.3758/s13428-016-0800-7 27573006

[16] Kroehne U, Goldhammer F. How to conceptualize, represent, and analyze log data from technology-based assessments? A generic framework and an application to questionnaire items. Behaviormetrika. 2018;45:527–63.

[17] Li T, et al. Smartphone app usage analysis: datasets, methods, and applications. IEEE Commun Surv Tutor. 2022;24:937–66.

[18] Investigating Country Differences in Mobile App User Behavior and Challenges for Software Engineering | IEEE Journals & Magazine | IEEE Xplore. Available from: https://ieeexplore.ieee.org/document/6913003

[19] Figueroa C, Johnson C, Verster A, Baggaley R. Attitudes and acceptability on HIV self-testing among key populations: a literature review. AIDS Behav. 2015;19(11):1949–65. doi: 10.1007/s10461-015-1097-8 26054390 PMC4598350

[20] Johnson C, Baggaley R, Forsythe S, van Rooyen H, Ford N, Napierala Mavedzenge S, et al. Realizing the potential for HIV self-testing. AIDS Behav. 2014;18 Suppl 4:S391-5. doi: 10.1007/s10461-014-0832-x 24986599

[21] Kirk GD, Himelhoch SS, Westergaard RP, Beckwith CG. Using mobile health technology to improve HIV care for persons living with HIV and substance abuse. AIDS Res Treat. 2013;2013:194613. doi: 10.1155/2013/194613 24381751 PMC3870121

[22] Sullivan PS, Jones J, Kishore N, Stephenson R. The roles of technology in primary HIV prevention for men who have sex with men. Curr HIV/AIDS Rep. 2015;12(4):481–8. doi: 10.1007/s11904-015-0293-5 26519083

[23] Wood OR, Garofalo R, Kuhns LM, Scherr TF, Zetina APM, Rodriguez RG, et al. A randomized controlled trial of an mHealth intervention for increasing access to HIV testing and care among young cisgender men and transgender women: the mLab App study protocol. BMC Public Health. 2021;21(1):1959. doi: 10.1186/s12889-021-12015-w 34715833 PMC8554516

[24] Schnall R, et al. Efficacy of the mLab App: a randomized clinical trial for increasing HIV testing uptake using mobile technology. J Am Med Inform Assoc. 2024. 10.1093/jamia/ocae261 PMC11756647 39560363

[25] Khasawneh N, Al-Salman R, Al-Hammouri AT, Conrad S. A generic framework for collecting and mining client paradata for web applications. JETWI. 2012;4(4):324–32. doi: 10.4304/jetwi.4.4.324-332

[26] Sanabria G, Scherr T, Garofalo R, Kuhns LM, Bushover B, Nash N, et al. Usability evaluation of the mLab App for improving home HIV testing behaviors in youth at risk of HIV infection. AIDS Educ Prev. 2021;33(4):312–24. doi: 10.1521/aeap.2021.33.4.312 34370566 PMC8487399

[27] Scherr TF, et al. Segmenting user behavior: a clustering approach to understanding digital health engagement. Rev.

[28] Scherr TF, Hardcastle A, Moore CP, Majji D, Kuhns LM, Garofalo R, et al. Detailed HIV self-testing patterns derived from paradata in the mLab App clinical trial. AIDS Behav. 2026. doi: 10.1007/s10461-025-05013-1 41511693 PMC13303770

[29] Asadzadeh A, Kalankesh LR. A scope of mobile health solutions in COVID-19 pandemics. Inform Med Unlocked. 2021;23:100558. doi: 10.1016/j.imu.2021.100558 33842688 PMC8019236

[30] Birnbaum F, Lewis D, Rosen RK, Ranney ML. Patient engagement and the design of digital health. Acad Emerg Med Off J Soc Acad Emerg Med. 2015;22(6):754–6. doi: 10.1111/acem.12692 25997375 PMC4674428

[31] Liang S, Wei Z, Zang L. An exploratory study of factors influencing user app abandonment on smartphones. Libr Hi Tech. 2024;43(2–3):622–37. doi: 10.1108/lht-07-2022-0349

[32] Greve M, Brendel AB, van Osten N, Kolbe LM. Overcoming the barriers of mobile health that hamper sustainability in low-resource environments. J Public Health (Berl). 2021;30(1):49–62. doi: 10.1007/s10389-021-01536-8

[33] Pang H, Ruan Y, Wang Y. Unpacking detrimental effects of network externalities on privacy invasion, communication overload and mobile app discontinued intentions: a cognition-affect-conation perspective. Behav Sci. 2023;13:47. 36661619 10.3390/bs13010047 PMC9855135

[34] Morris JW, Morris A. App-ed out: logics of success and failure in app stores. Comput Cult. 2019. Available from: http://computationalculture.net/app-ed-out-logics-of-success-and-failure-in-app-stores/

[35] Hiniker A, Patel SN, Kohno T, Kientz JA. Why would you do that? predicting the uses and gratifications behind smartphone-usage behaviors. Proceedings of the 2016 ACM International Joint Conference on Pervasive and Ubiquitous Computing. New York, NY, USA: Association for Computing Machinery; 2016. p. 634–45. doi: 10.1145/2971648.2971762

[36] Scherr TF, Hardcastle AN, Moore CP, DeSousa JM, Wright DW. Understanding on-campus interactions with a semiautomated, barcode-based platform to augment COVID-19 contact tracing: app development and usage. JMIR Mhealth Uhealth. 2021;9(3):e24275. doi: 10.2196/24275 33690142 PMC8006900

[37] Brands of smartphones owned by Americans 2022, by income. Statista. https://www.statista.com/statistics/512863/smartphones-cell-phones-tablets-and-ereaders-brands-owned-by-affluent-americans/

[38] User-Agent - HTTP | MDN; 2024. Available from: https://developer.mozilla.org/en-US/docs/Web/HTTP/Headers/User-Agent

[39] A classification of web browser fingerprinting techniques | IEEE Conference Publication | IEEE Xplore. Available from: https://ieeexplore.ieee.org/abstract/document/7266460

[40] Obermeyer CM, Baijal P, Pegurri E. Facilitating HIV disclosure across diverse settings: a review. Am J Public Health. 2011;101:1011–23. 21493947 10.2105/AJPH.2010.300102 PMC3093267

[41] Zhao S, Li S, Ramos J, Luo Z, Jiang Z, Dey AK, et al. User profiling from their use of smartphone applications: a survey. Pervasive Mob Comput. 2019;59:101052. doi: 10.1016/j.pmcj.2019.101052

