# Supplementary material for: Exploring digital health user engagement: General app usage patterns from a clinical trial with the mLab App
Source: PLOS Digit Health. 2026 Jun 25;5(6):e0001452. doi: 10.1371/journal.pdig.0001452 (PMC13298777; doi:10.1371/journal.pdig.0001452)
Supplement: S1 Fig — A. Landing page. B. Preview test page. (DOCX) [file pdig.0001452.s001.docx]

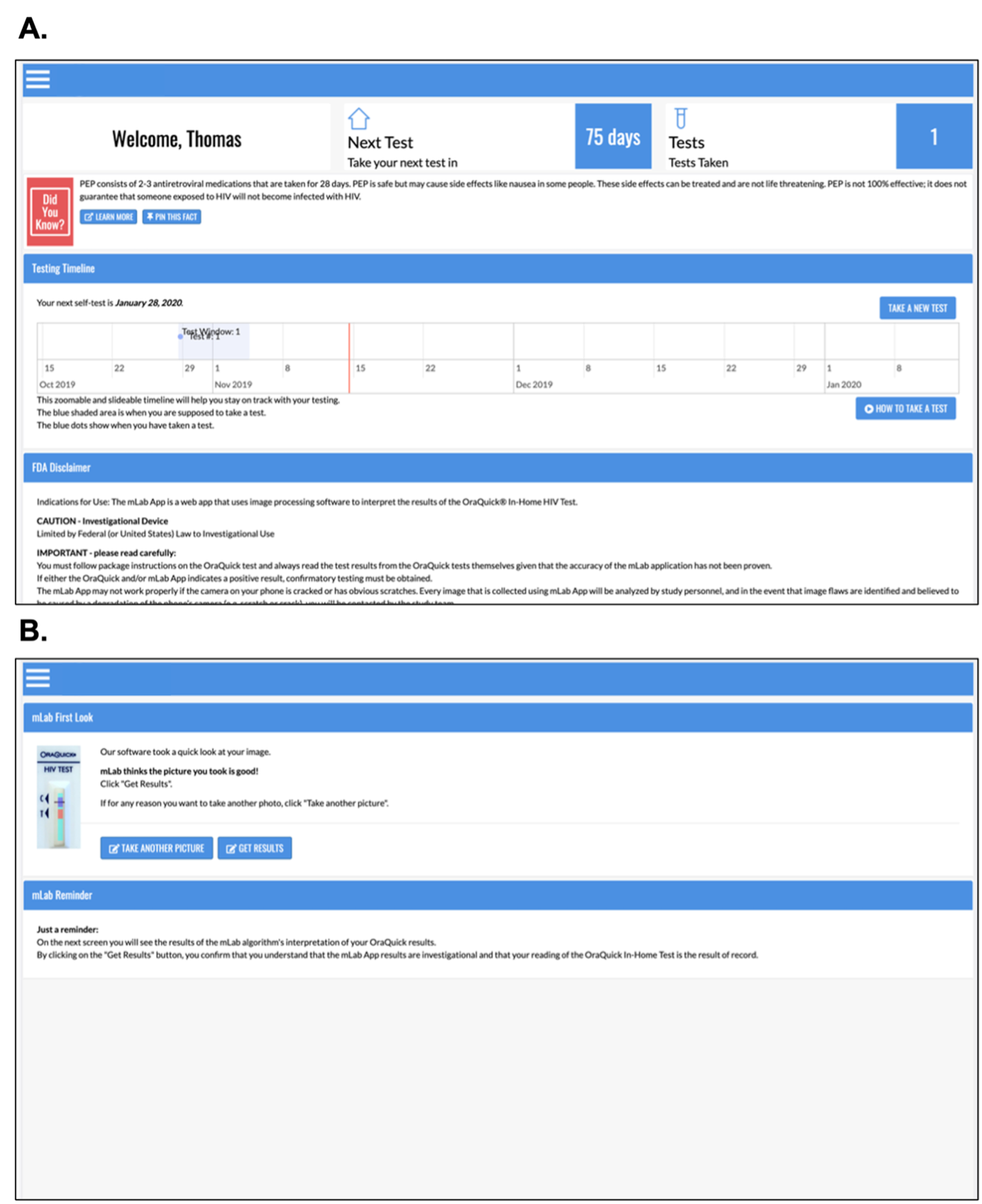


**S1 Fig.** Representative screenshots of the desktop layout of the mLab App. **A.** Landing page. **B.** Preview test page.
