# Supplementary figures and images for: Exploring digital health user engagement: General app usage patterns from a clinical trial with the mLab App
Source: PLOS Digit Health. 2026 Jun 25;5(6):e0001452. doi: 10.1371/journal.pdig.0001452 (PMC13298777; doi:10.1371/journal.pdig.0001452)

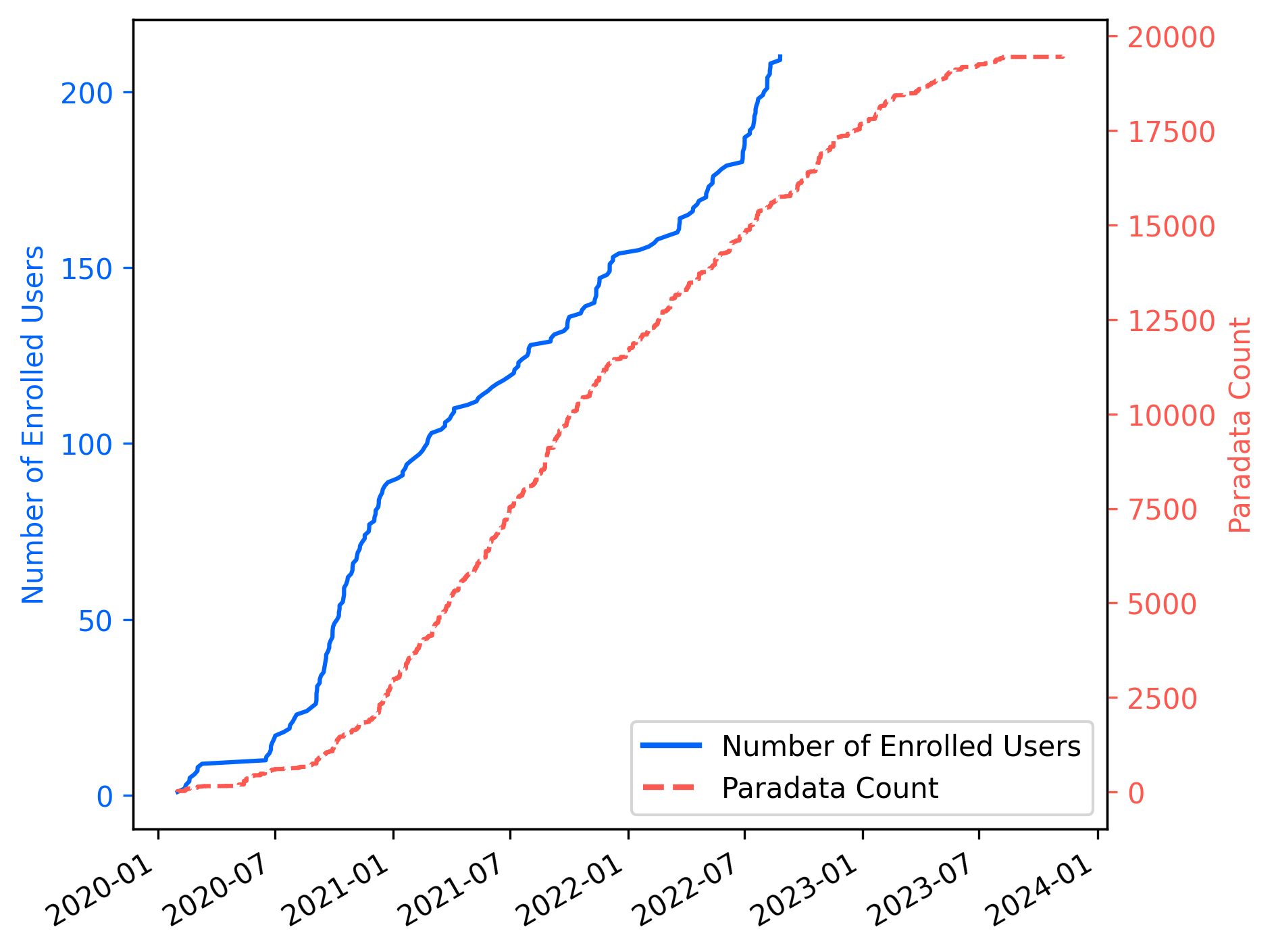


**S2 Fig.** User enrollment and total paradata count throughout the duration of the study.

Supplement: S2 Fig — (DOCX) [file pdig.0001452.s002.docx]
