# Supplementary material for: Exploring digital health user engagement: General app usage patterns from a clinical trial with the mLab App
Source: PLOS Digit Health. 2026 Jun 25;5(6):e0001452. doi: 10.1371/journal.pdig.0001452 (PMC13298777; doi:10.1371/journal.pdig.0001452)
Supplement: S3 Fig — A. Number of sessions by day of the week. B. Number of sessions by hour of the day. C. Number of tests by day of the week. D. Number of tests by hour of the day. (DOCX) [file pdig.0001452.s003.docx]

**
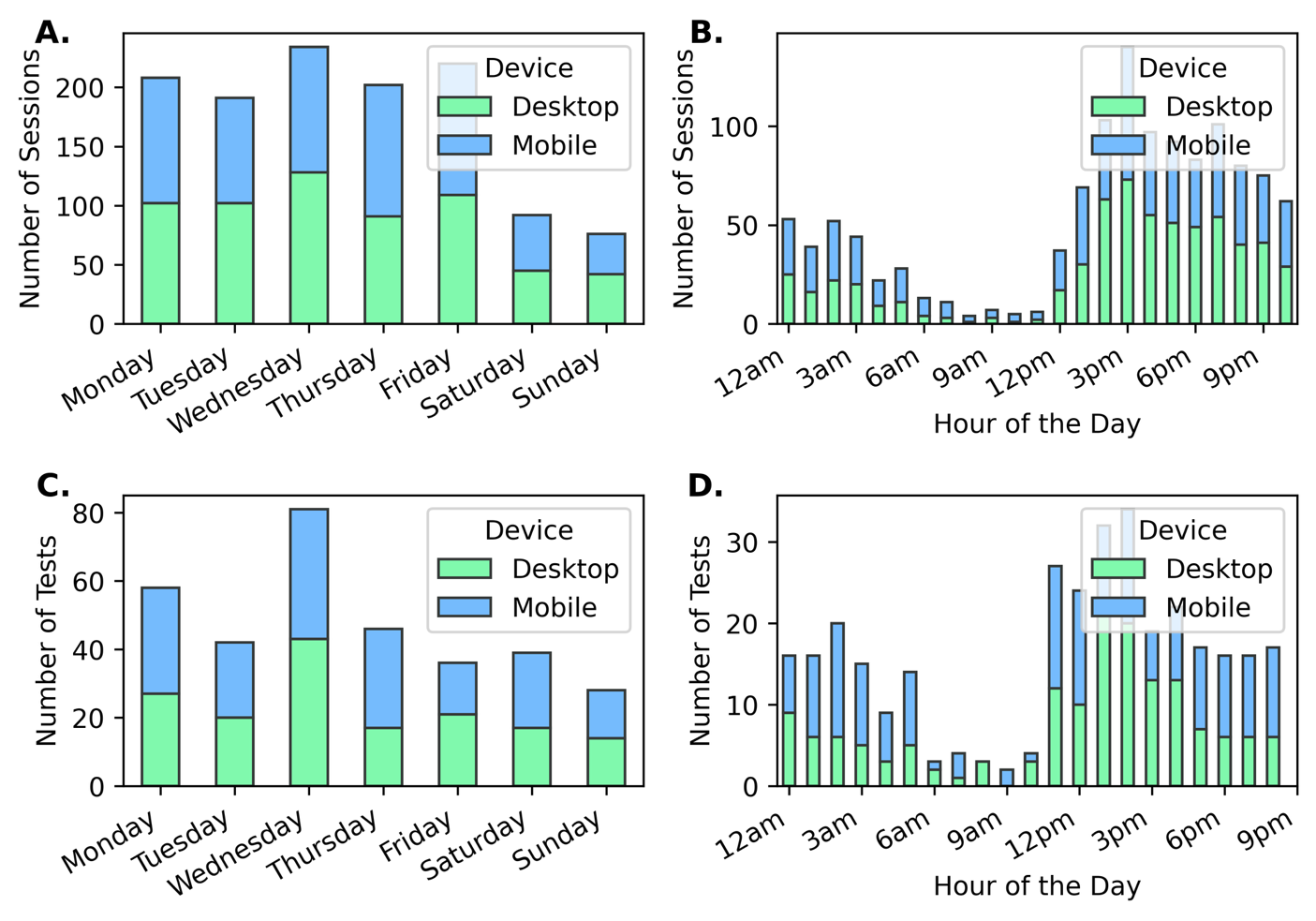
**

**S3 Fig.** Temporal counts of sessions and tests, stacked by device type. **A.** Number of sessions by day of the week. **B.** Number of sessions by hour of the day. **C.** Number of tests by day of the week. **D**. Number of tests by hour of the day.
