# Supplementary material for: Exploring digital health user engagement: General app usage patterns from a clinical trial with the mLab App
Source: PLOS Digit Health. 2026 Jun 25;5(6):e0001452. doi: 10.1371/journal.pdig.0001452 (PMC13298777; doi:10.1371/journal.pdig.0001452)
Supplement: S4 Fig — (DOCX) [file pdig.0001452.s004.docx]

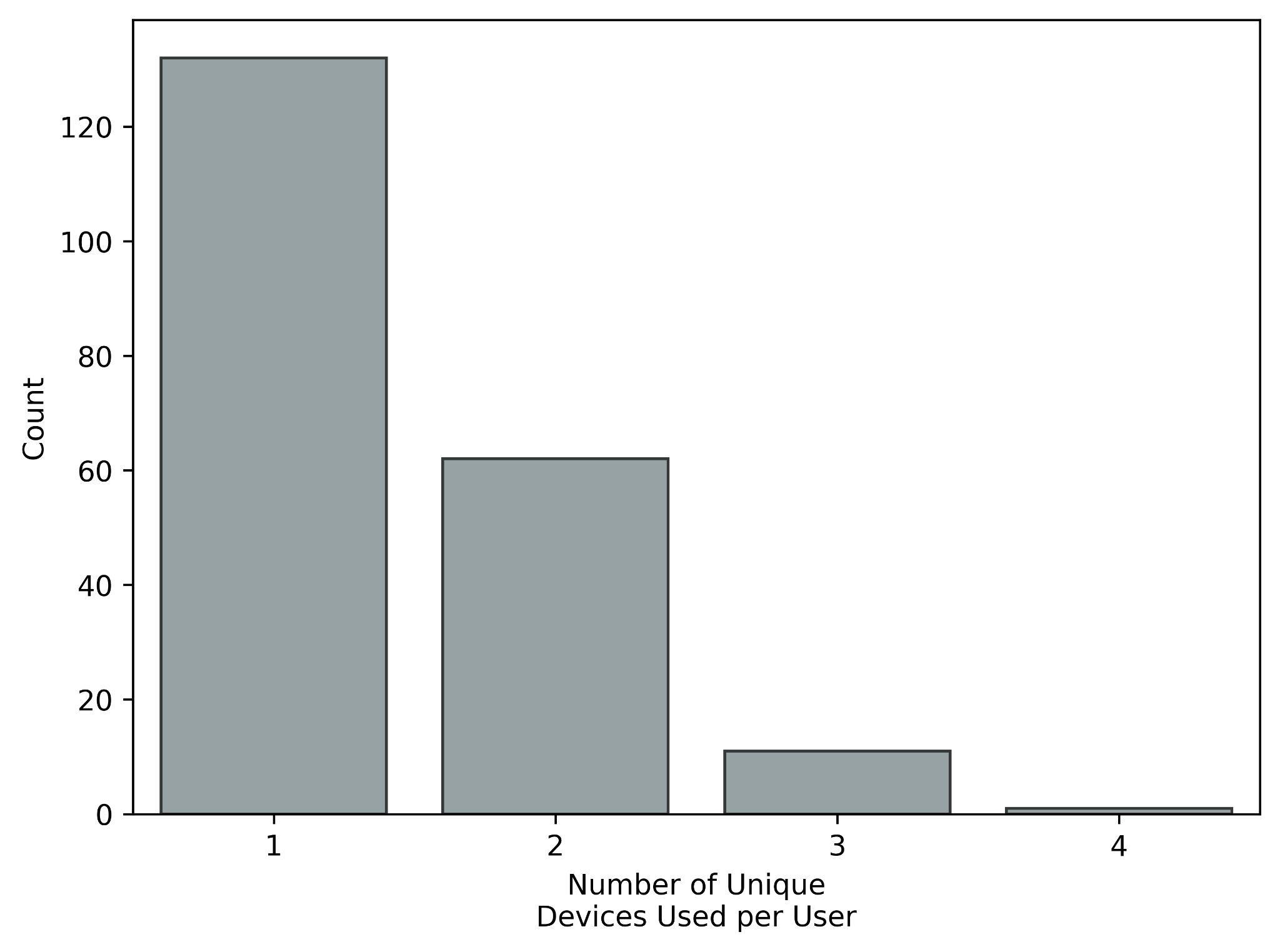


**S4 Fig.** Number of unique devices each user used, per the browser agent of the first paradata entry of each session.
