# Supplementary material for: Exploring digital health user engagement: General app usage patterns from a clinical trial with the mLab App
Source: PLOS Digit Health. 2026 Jun 25;5(6):e0001452. doi: 10.1371/journal.pdig.0001452 (PMC13298777; doi:10.1371/journal.pdig.0001452)
Supplement: S6 Fig — The navigation pathway is scaled to 100% along the current-page dimension (actual counts are shown in parentheses). For any given row, each column shows the percentage of times that screen was the next screen, from the row’s current page, in a user’s navigation history. (DOCX) [file pdig.0001452.s006.docx]

**
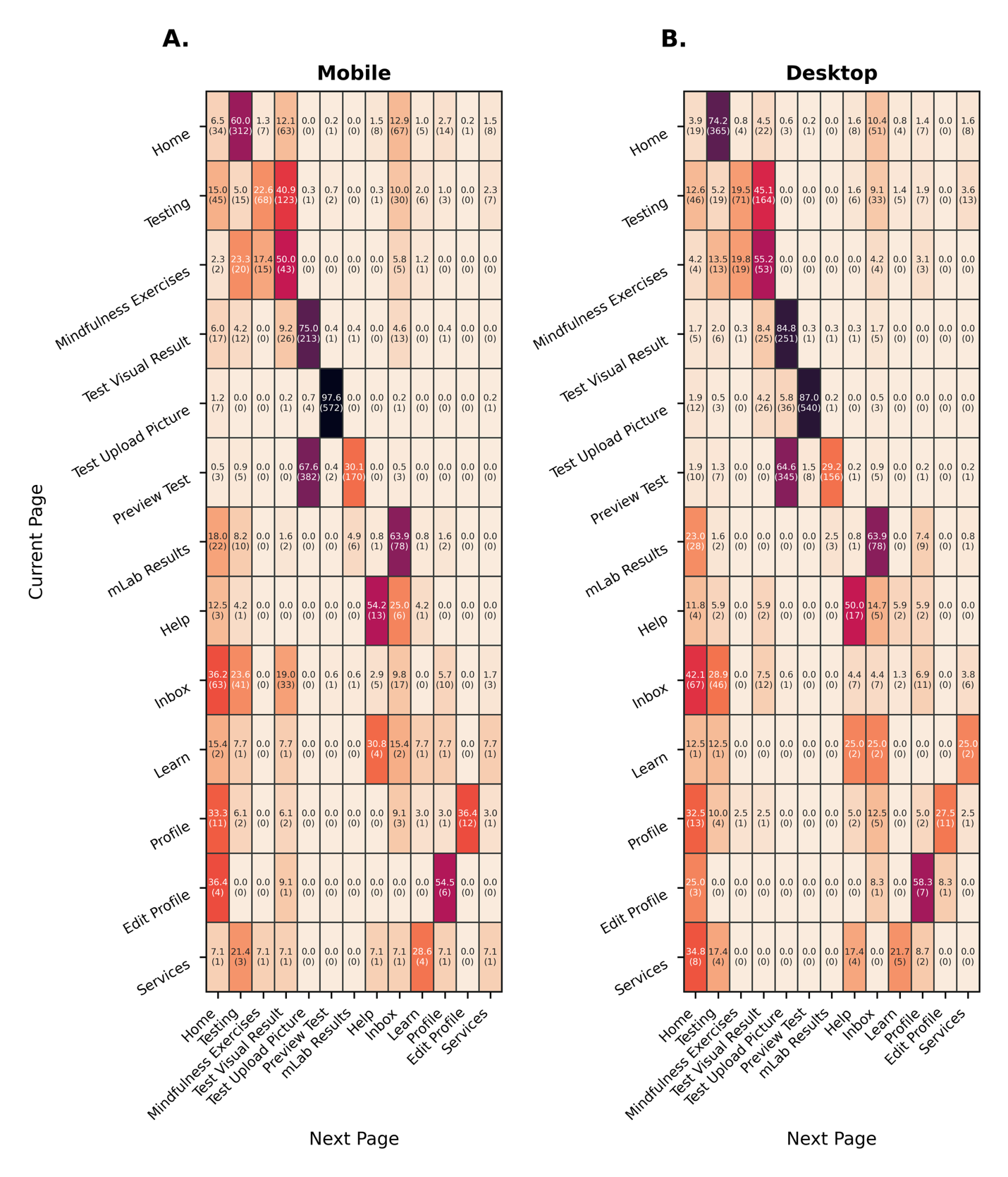
**

**S6 Fig.** Heat maps of navigation pathways, from the current page (y-axis) to the next page (x-axis), isolated by (A) mobile and (B) desktop sessions. The navigation pathway is scaled to 100% along the current-page dimension (actual counts are shown in parentheses). For any given row, each column shows the percentage of times that screen was the next screen, from the row’s current page, in a user’s navigation history.
