# Supplementary material for: Exploring digital health user engagement: General app usage patterns from a clinical trial with the mLab App
Source: PLOS Digit Health. 2026 Jun 25;5(6):e0001452. doi: 10.1371/journal.pdig.0001452 (PMC13298777; doi:10.1371/journal.pdig.0001452)
Supplement: S7 Fig — (DOCX) [file pdig.0001452.s007.docx]

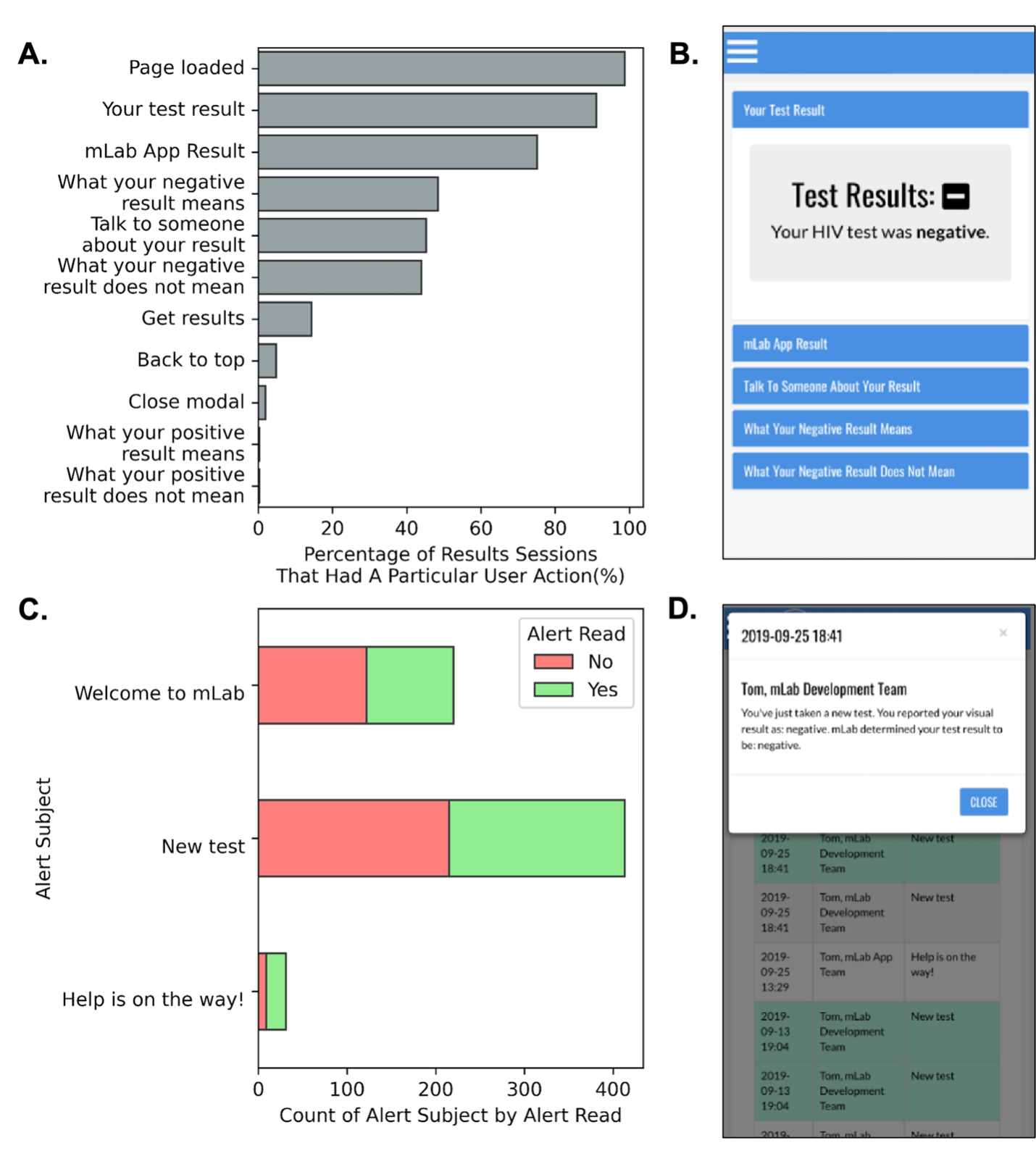
**S7 Fig.** **A.** The percentage of sessions that reached the results page where a user performed a specific action on **B.** the Results page. **C.** The percentage of alerts that were read by participants in **D.** their mLab inbox.
